# *M. tuberculosis* infection of human iPSC-derived macrophages reveals complex membrane dynamics during xenophagy evasion

**DOI:** 10.1242/jcs.252973

**Published:** 2020-11-25

**Authors:** Elliott M. Bernard, Antony Fearns, Claudio Bussi, Pierre Santucci, Christopher J. Peddie, Rachel J. Lai, Lucy M. Collinson, Maximiliano G. Gutierrez

**Affiliations:** 1 Host-Pathogen Interactions in Tuberculosis Laboratory, The Francis Crick Institute, 1 Midland Road, London, NW1 1AT, UK; 2 Electron Microscopy Science Technology Platform, The Francis Crick Institute, 1 Midland Road, London, NW1 1AT, UK; 3 Department of Medicine, Imperial College London, London W2 1PG, UK

**Keywords:** Macrophage, Autophagy, Stem cell, Xenophagy, Tuberculosis

## Abstract

Xenophagy is an important cellular defence mechanism against cytosol-invading pathogens, such as *Mycobacterium tuberculosis* (Mtb). Activation of xenophagy in macrophages targets Mtb to autophagosomes; however, how Mtb is targeted to autophagosomes in human macrophages at a high spatial and temporal resolution is unknown. Here, we use human induced pluripotent stem cell-derived macrophages (iPSDMs) to study the human macrophage response to Mtb infection and the role of the ESX-1 type VII secretion system. Using RNA-seq, we identify ESX-1-dependent transcriptional responses in iPSDMs after infection with Mtb. This analysis revealed differential inflammatory responses and dysregulated pathways such as eukaryotic initiation factor 2 (eIF2) signalling and protein ubiquitylation. Moreover, live-cell imaging revealed that Mtb infection in human macrophages induces dynamic ESX-1-dependent, LC3B-positive tubulovesicular autophagosomes (LC3-TVS). Through a correlative live-cell and focused ion beam scanning electron microscopy (FIB SEM) approach, we show that upon phagosomal rupture, Mtb induces the formation of LC3-TVS, from which the bacterium is able to escape to reside in the cytosol. Thus, iPSDMs represent a valuable model for studying spatiotemporal dynamics of human macrophage–Mtb interactions, and Mtb is able to evade capture by autophagic compartments.

## Abstract (precis)

**Summary:** Following phagosome membrane damage, *Mycobacterium tuberculosis* induces the formation of, and escapes from, tubulovesicular autophagosomes in human induced pluripotent stem cell-derived macrophages.

## INTRODUCTION

Macrophages are arguably the most important cell type during infection with *M. tuberculosis* (Mtb), one of the most successful bacterial pathogens globally. They are one of the first cells infected, provide a reservoir for bacterial replication and mediate bacterial dissemination (Cohen et al., 2018). Mtb is unable to establish an effective infection in mice lacking alveolar macrophages, further implicating them as playing a key role in tuberculosis (TB) pathogenesis (Leemans et al., 2001). As well as providing a host for Mtb, macrophages are crucial for the initiation and maintenance of granulomas, a hallmark of human TB (Davis and Ramakrishnan, 2009; Davis et al., 2002). Additionally, antigen presentation by macrophages stimulates the release of IFN-γ from primed T cells to enhance control of the infection (Flynn and Chan, 2001).

Following phagocytosis by macrophages, Mtb initially resides within phagosomes before damaging the phagosomal membrane to access the cytosol. This is achieved through the co-action of the type VII secretion system ESX-1, encoded within the virulence-related RD1 region, and the presence of cell wall-associated phthiocerol dimycocerosates (PDIM) lipids (Augenstreich et al., 2017; Barczak et al., 2017; Lerner et al., 2017, 2018; Quigley et al., 2017; van der Wel et al., 2007). Cytosolic access is important for selective recognition of Mtb by the autophagy machinery and induction of immune responses driven by type I interferon (IFN) (Watson et al., 2015).

The selective targeting of bacteria to the autophagy pathway, known as xenophagy, is important for control of many bacterial pathogens, including group A *Streptococcus* and *Salmonella* (Nakagawa et al., 2004; Thurston et al., 2009). These pathogens, among others, are targeted to xenophagy as they attempt to access the cytosol through damaging the pathogen-containing vacuole. This is achieved through the action of galectins and ubiquitin recruiting the autophagy initiation machinery and tethering the forming autophagosome to the pathogen (Perrin et al., 2004; Ravenhill et al., 2019; Thurston et al., 2012). Mtb is targeted to autophagosomes through ubiquitination by the E3 ligases Parkin and SMURF1 (Franco et al., 2016; Manzanillo et al., 2013). In mouse macrophages, autophagy induction restricts bacterial replication; however, rapamycin treatment of human macrophages enhances Mtb growth (Andersson et al., 2016; Gutierrez et al., 2004). Despite being clear that Mtb is targeted to autophagosomes, there are no studies examining the dynamics of autophagic targeting of Mtb in macrophages by live-cell imaging so several questions remain to be answered. For example, is Mtb able to escape from autophagosomes? In addition, what is the fate of the membrane remnants resulting from phagosome lysis?

ESX-1 is one of five type VII secretion systems encoded by the Mtb genome. The loss of the region of difference 1 (RD1) locus, which encodes many of the ESX-1 components, is largely responsible for the attenuation of *M. bovis* that led to the vaccine strain *M. bovis* BCG (Pym et al., 2002). As well as being essential for successful phagosomal rupture and cytosolic access, ESX-1 is required for the release of Mtb DNA into the macrophage cytosol to activate the cGAS/STING/TBK1 axis for type I IFN production and xenophagy activation (Watson et al., 2012, 2015). Identification of all ESX-1 substrates and their functions, as well as how secretion is achieved by type VII section systems are important questions that remain unanswered.

Owing to the essential role macrophages play in TB pathogenesis, the interaction between Mtb and macrophages has been widely investigated; primarily in mouse macrophages, including RAW264.7 cells and primary bone marrow-derived macrophages (BMDMs). Although these cells provide a useful model system, there are fundamental differences between the human and mouse macrophage responses to Mtb infection. Mouse macrophages produce high, bactericidal levels of nitric oxide (NO) *in vitro* whereas there are varying reports of human macrophages producing relatively low levels of NO (Chan et al., 1995; Jung et al., 2013). Additionally, human macrophages show different transcriptional responses following activation of pattern recognition receptors, such as TLR4, when compared to mouse macrophages (Schroder et al., 2012). Moreover, the subcellular distribution of virulent Mtb in mouse and human macrophages is different. Mtb is able to localise in the cytosol of human macrophages as early as 4 h post infection (Lerner et al., 2017), whereas in BMDM Mtb primarily remains within membrane-bound compartments (Schnettger, 2016). Finally, in mice the xenophagy adaptor NDP52 (also known as CALCOCO2), which is necessary for *in situ* phagophore biogenesis (Ravenhill et al., 2019; Vargas et al., 2019), lacks a C-terminal zinc finger domain that binds ubiquitin and Galectin 8. Thus, there are potential differences in how Mtb is targeted to autophagosomes between mouse and human macrophages (Tumbarello et al., 2015).

Our understanding of human macrophage cell biology has been hampered by the lack of scalable models. For many *in vitro* studies with human macrophages, primary human monocyte derived macrophages (hMDMs) are obtained from the CD14-positive monocyte fraction in the blood and are subsequently differentiated into macrophages with either M-CSF or GM-CSF (Lerner et al., 2017). Although this system provides a wealth of cells for investigations and likely recapitulates the interactions that occur between Mtb and recruited macrophages, their ability to reflect the tissue-resident alveolar macrophages, which show different responses to Mtb infection (Pisu et al., 2020), is unclear and donor-to-donor variability can confound results. Additionally, these human monocyte-derived macrophages are not genetically tractable – they are difficult to transfect and CRISPR/Cas9-mediated gene knockout has only been reported once (Hiatt et al., 2020 preprint). One alternative is the differentiation of the immortalised monocytic cell line THP-1 with phorbol esters to produce macrophages. This line has several limitations, including large genomic rearrangements (Odero et al., 2000) and the activation status of the cells (Bussi and Gutierrez, 2019). In this context, human induced pluripotent stem cells (iPSCs) are revolutionising models for probing biological questions in multiple fields, as well as being hailed as future treatments for a multitude of diseases with the potential to more accurately reflect *in vivo* human cell types (Hyman and Simons, 2011; Zhang and Reilly, 2017). Several protocols have now been published for the generation of macrophages from iPSCs (iPSDMs) (Lee et al., 2018). These iPSDMs have similar ontogeny to yolk sack-derived tissue-resident macrophages (Buchrieser et al., 2017) with further potential to derive tissue-specific macrophages through co-culture setups or *in vivo* organ transfer (Haenseler et al., 2017; Takata et al., 2017). Additionally, iPSDMs have been shown to respond to immunological stimuli, such as lipopolysaccharide (LPS), in a manner akin to hMDMs in side-by-side transcriptomic and functional comparisons (Alasoo et al., 2015; Cao et al., 2019; Lin et al., 2016; Zhang et al., 2015).

In this work, we validate an embryonic body-derived iPSDM model as a tool for studying the cell biology of host–pathogen interactions in TB. We use this model to probe the transcriptional response of human macrophages to virulent Mtb (Mtb WT) and the attenuated mutant lacking the ESX-1 secretion system (Mtb ΔRD1). Strikingly, the response of iPSDMs to Mtb showed significant differences between WT and ΔRD1 only at 48 h whereas no differences were observed 2 h post infection. We further investigated the spatiotemporal dynamics of autophagic targeting of Mtb and revealed a remarkable ESX-1-mediated, phagosome membrane damage driven re-localisation of LC3B (also known as MAP1LC3B) to tubulovesicular structures (LC3-TVS) in the vicinity of the bacilli and the ability of Mtb to escape from LC3-TVS into the cytosol. Correlative live-cell and focused ion beam scanning electron microscopy (FIB SEM) of LC3B-positive structures uncovered an underlying membrane complexity as well as Mtb–macrophage organelle interactions. Taken together, we show here that iPSDMs represent a novel *in vitro* approach to interrogate questions in the context of the cell autonomous response of human macrophages to Mtb.

## RESULTS

### Generation, differentiation and characterisation of human iPSDMs

In order to better understand the role of xenophagy of Mtb in human macrophages, we established an embryonic body (EB)-based protocol for iPSDM differentiation where haematopoiesis is induced under serum-free conditions (van Wilgenburg et al., 2013). When selecting the iPSC lines to generate iPSDMs, we paid special attention to mutations that might affect xenophagy, since most of the commonly used iPSCs available contain mutations in the Atg16L1 gene (E.M.B., unpublished observations), which are common in the population, and important for xenophagic responses (Lassen et al., 2014). Based on these criteria, we selected the iPSC line eika_2 (hPSCreg name: WTSIi002-A) from the Wellcome Trust Sanger Institute for our studies. iPSDMs were differentiated from EIKA2 iPSCs following the protocol established in Fig. S1A. Next, iPSDMs were characterised by flow cytometry for surface expression of macrophage markers. After M-CSF-induced differentiation, iPSDMs showed a reduction in CD14 and an increase in CD11b (also known as ITGAM) surface expression levels (Fig. S1B). Macrophages stimulated with interferon-γ (IFN-γ) and LPS displayed lower surface expression of the IFN-γ receptor CD119 and increased expression of the T cell stimulator CD86 as previously reported for hMDMs (Schulz et al., 2019). Upon differentiation, the cells downregulated the surface expression of the phagocytic receptors CD163, CD169 and CD206 (Fig. S1B). Moreover, RNA-seq analysis showed the presence of common macrophage specific transcription factors *PPARG*, *ID2*, *RUNX3*, *MEF2C* and *ID3* (Lee et al., 2018). When this was compared to previous AmpliSeq data (Papp et al., 2018) there was a significant overlap in the transcription factors detected, especially when compared with hMDMs (Fig. S1C–E). We were also able to detect transcripts of well-described macrophage markers, including those examined by flow cytometry in Fig. S1B. Importantly, the presence of stem cell marker reads was minimal (Fig. S1F). Functionally, the iPSDMs were able to phagocytose Mtb as shown by SEM (Fig. S1G). Moreover, the expression of indoleamine-dioxygenase (IDO) and inducible nitric oxide synthase (iNOS) was increased after IFN-γ and LPS stimulation (Fig. S1H). Despite this, NO production was not detected in the supernatants (Fig. S1I). Altogether, the established protocol and analysis provide strong evidence for the use of iPSDMs as a relevant *in vitro* model that reflect well-described aspects of human primary macrophages.

### Mtb is dynamically localised in membrane-bound compartments and the cytosol of iPSDMs

**Fig. 1.**
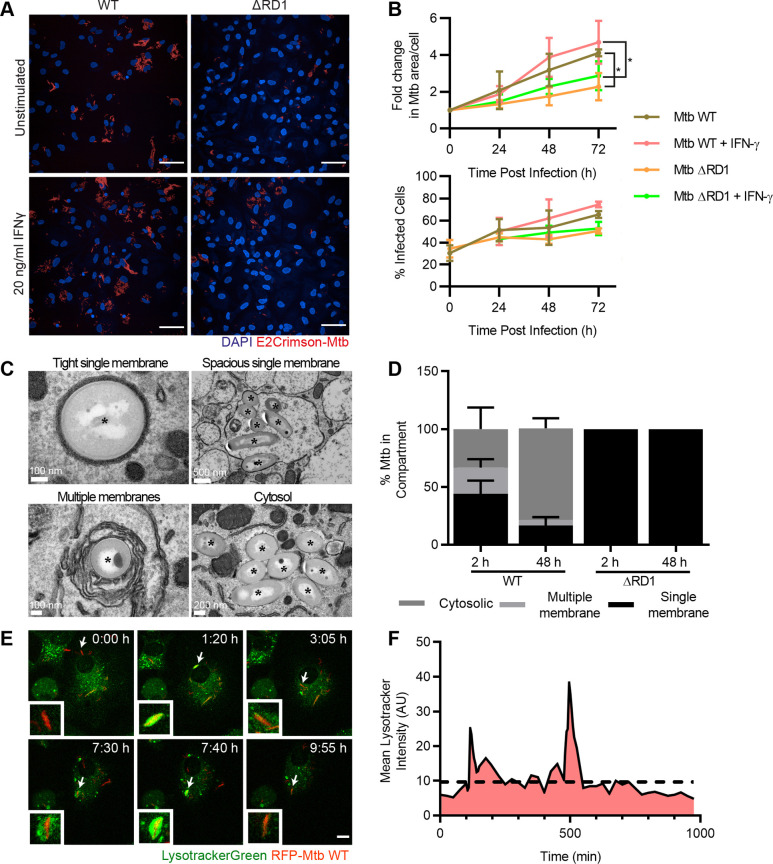
**iPSDMs as a model to investigate Mtb–human macrophage interactions.** (A) Representative maximum projection confocal fluorescence images showing E2Crimson-Mtb WT or ΔRD1 (red) in human iPSDMs at 48 h post infection with or without exogenous IFN-γ stimulation. Scale bars: 50 µm. (B) Top graph shows quantification of fold change, relative to time 0, in mean Mtb area per infected cell from micrographs represented in A. Data are mean±s.d. from three biological replicates. *P<0.05 (Holm–Sidak's multiple comparisons test). Bottom graph shows the percentage of infected cells as mean±s.d. from the same three biological replicates. (C) Representative transmission electron micrographs of Mtb WT in distinct subcellular locations. Asterisks indicate bacteria. (D) Stereological quantification of the subcellular distribution of Mtb WT and ΔRD1 from TEM images. Data are means±s.d. from two independent experiments with a minimum of 49 infected cells analysed per condition. (E) Maximum projection stills from Movie 1 showing iPSDMs stained with Lysotracker Green DND-26 and infected with RFP-Mtb WT. Arrows highlight bacterium of interest shown in inset. Frames acquired every 5 min. Scale bar: 10 µm. Images filtered with a Gaussian blur, radius 0.8. (F) Quantification of Lysotracker association to Mtb shown in C, dashed line indicates cut off for negative bacteria. AU, arbitrary units.

We next sought to investigate the suitability of iPSDMs as a model for the study of Mtb infection. iPSDMs were efficiently infected with wild-type Mtb (Mtb WT) or Mtb lacking the RD1 genomic region that encodes for the ESX-1 type VII secretion system (Mtb ΔRD1), both expressing the fluorescent protein E2Crimson (Fig. 1A). At 2 h post infection, ∼30% of cells were infected, with this steadily increasing over 72 h of infection (Fig. 1B). We found that Mtb efficiently replicates in iPSDMs in an RD1-dependent manner and addition of exogenous IFN-γ during the infection led to a slight increase in the replication of Mtb WT and ΔRD1 by 48 h post infection (Fig. 1B). In hMDMs, Mtb is known to localise in diverse intracellular environments including phagosomes, autophagosomes and the cytosol (Bussi and Gutierrez, 2019; Lerner et al., 2017). Similarly, in iPSDMs, we found that Mtb localised to multiple environments, including those surrounded by spacious and tight single membranes or multiple membranes, as well as in the cytosol (Fig. 1C). Stereological analysis of transmission electron micrographs of infected iPSDMs revealed that 33.2±25.9% (mean±s.d.) of Mtb WT localised freely in the cytosol, without any visible host membranes around the bacillus, at 2 h post infection (Fig. 1D). By 48 h, Mtb WT was primarily localised in the cytosol, with a minor fraction localised in multiple membrane- and single membrane-bound compartments (Fig. 1D). In contrast, Mtb ΔRD1 remained localised to membrane-bound compartments up to 48 h after infection (Fig. 1D). In order to better understand the dynamics of membrane-bound versus cytosolic localisation, we used Lysotracker Green (LTR) as a probe for Mtb phagosome membrane integrity (Repnik et al., 2017; Schnettger et al., 2017). LTR is irreversibly protonated in the endolysosomal lumen preventing its diffusion across the membrane and trapping it within intact endolysosomes, thus for the probe to be released from the lumen, the lysosomal membrane integrity must be compromised. Mtb phagosomes showed a rapid leak of LTR followed by recovery of the LTR signal, suggesting that transient events of Mtb phagosome membrane damage are followed by either repair or recapture of Mtb from the cytosol into intact compartments (Fig. 1E,F; Movie 1). Moreover, some bacilli showed loss of LTR not followed by recovery in the timeframes imaged (data not shown). We conclude that, similar to what is found for hMDMs (Lerner et al., 2017; van der Wel et al., 2007), Mtb dynamically localises in phagosomes as well as in the cytosol of iPSDMs in an RD1-dependent manner.

### Gene expression analysis reveals a differing inflammatory response of iPSDMs after Mtb WT or Mtb ΔRD1 infection

**Fig. 2.**
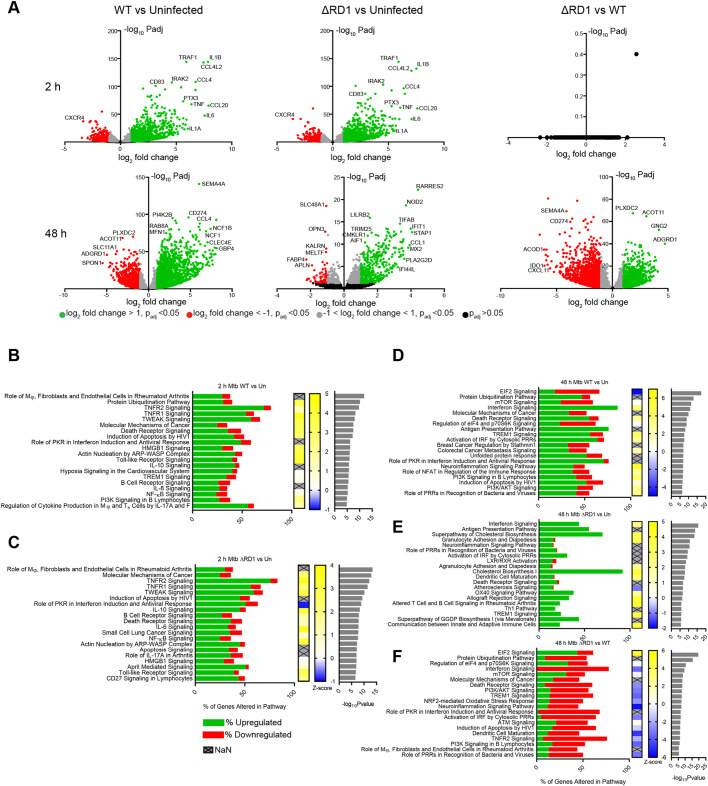
**RD1-dependent transcriptional responses in iPSDMs.** (A) Volcano plots of differentially expressed genes from RNA-seq analysis of iPSDMs infected with either RFP–Mtb WT or Mtb ΔRD1 for 2 or 48 h. Green spots show genes ≥2-fold over-expressed, *P*<0.05. Red spots show genes ≥2-fold under-expressed, *P*<0.05. Grey spots show genes <2 fold over- or under-expressed *P*<0.05. Black spots show genes expressed with no significant difference between conditions, *P*>0.05. Selected genes are labelled. (B,C) Top 20 pathways by *P*-value from IPA analysis of differentially expressed genes from 2 h infected samples comparing Mtb WT infected versus uninfected (B) and Mtb RD1 infected versus uninfected (C). (D–F) The top 20 pathways by *P*-value from IPA analysis of differentially expressed genes from RNA-seq shown in Fig. 3 from (D) Mtb WT infected versus uninfected 48 h, (E) Mtb ΔRD1 infected versus uninfected 48 h and (F) Mtb ΔRD1 infected versus Mtb WT infected 48 h comparisons. *P*-value cut off for differentially expressed genes for pathway analysis was *P*<0.01. Bar charts show percentage of genes altered in each pathway, green shows upregulated, red downregulated genes. Heat map shows computed pathway activation by Z score computed by IPA, grey boxes with an ‘X’ indicate activation state could not be predicted. Right hand bar charts indicate –log_10_ *P*-values.

We then characterised the transcriptional response of iPSDMs to infection. After 2 h of infection with Mtb WT, there was a significant upregulation of immune modulators such as *TNF*, *IL-1A*, *IL-1B* and *IL-6* (Fig. 2A). A small group of genes were significantly downregulated, including *CXCR4* (Fig. 2A). A similar pattern was observed after Mtb ΔRD1 infection (Fig. 2A). Remarkably, when the iPSDM populations infected with Mtb WT and ΔRD1 were compared, there were no significant differences in gene expression, suggesting a common response of iPSDM early after infection (Fig. 2A). At 48 h, Mtb ΔRD1-infected cells showed lower expression of important immune modulators involved in modulating T cell responses such as *IDO1*, *CXCL11*, *CD274* and *SEMA4A* comparted to Mtb WT-infected iPSDMs (Fig. 2A). Ingenuity pathway analysis (IPA) revealed that at 2 h after infection the main transcriptional response to both Mtb WT and Mtb ΔRD1 was inflammation and immune pathway activation (Fig. 2B,C). These data are consistent with previous transcriptional analysis of hMDM and human alveolar macrophages after Mtb infection (Papp et al., 2018). At 48 h after infection, Mtb WT infection upregulated genes involved in protein ubiquitylation (Fig. 2D), a pathway not significantly altered in Mtb ΔRD1-infected cells (Fig. 2E). Interestingly, Mtb ΔRD1-infected cells showed strong representation of pathways associated with altered lipid metabolism such as cholesterol biosynthesis, LXR/RXR activation and atherosclerosis signalling (Fig. 2E). At 48 h of infection, Mtb ΔRD1 infection did not significantly upregulate IFN, TREM1 and TNFR2 signalling (Fig. 2F), and there were fewer dysregulated genes in immune pathways after Mtb ΔRD1 infection when compared to Mtb WT (Fig. 2F). Taken together, this transcriptomic analysis shows that iPSDMs respond to Mtb infection by activating key immune defence pathways in a way that mimics previous studies in human macrophages (Papp et al., 2018). One key difference between iPSDMs infected with Mtb WT or Mtb ΔRD1 at 48 h was the activity of immune response pathways (Fig. S2A). In particular, pro-inflammatory cytokines associated with the immune response to TB were upregulated after 2 h of infection with both Mtb WT and Mtb ΔRD1. However, at 48 h only Mtb WT-infected cells retained this increased expression of pro-inflammatory cytokines (Fig. S2B). In agreement with the gene expression data, infection with Mtb WT significantly increased the secretion of IFN-α, TNF-α, IL-8, MCP-1, IL-1-α, IL-6 and MIP1-α and MIP1-β (Fig. S2C). There was a clear trend of lower levels of secretion for these cytokines after infection with Mtb ΔRD1, which in some cases was entirely dependent on the RD1 region, as shown for IFN-α (Fig. S2C). Altogether, our cytokine profile analysis indicates a robust secretion of pro-inflammatory cytokines by iPSDMs after infection. Importantly, in iPSDM, type I IFN secretion was entirely dependent on the ESX-1 system as shown in several other *in vitro* cellular systems of Mtb infection (Stanley et al., 2007; Wassermann et al., 2015).

**Fig. 3.**
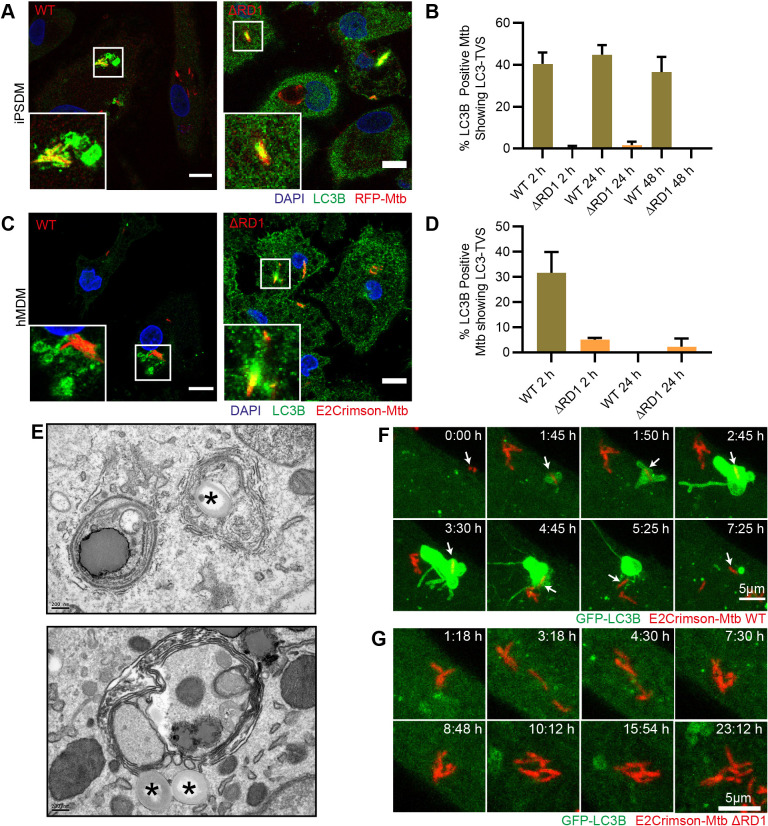
**Mtb induces formation of LC3-TVS in an RD1-dependent manner.** (A) Confocal fluorescence imaging of endogenous LC3B in iPSDMs infected with Mtb WT or ΔRD1 48 h post infection. Scale bars: 10 µm. Image representative of three biological replicates. (B) Manual quantification of the percentage of LC3B-positive bacteria from images represented in A that show a staining pattern with enlarged LC3 vesicles adjacent to the bacteria. Data are mean±s.e.m. from three biological replicates. (C) Confocal fluorescence imaging of endogenous LC3B in M-CSF-differentiated hMDMs infected with Mtb WT or ΔRD1 2 h post infection. Images representative of two biological replicates with two donors. Scale bars: 10 µm. (D) Manual quantification of the percentage of LC3B-positive bacteria from images represented in C that show a staining pattern with enlarged LC3B vesicles adjacent to the bacteria. Data are mean±s.e.m. from two biological replicates. (E) Transmission electron micrographs of Mtb WT-infected iPSDMs at 48 h post infection showing juxtaposition of Mtb and multilamellar structures containing cytosolic content. Asterisks indicate Mtb. Scale bars: 200 nm. (F,G) Maximum projection stills from live-cell imaging in Movie 2 (F) or Movie 3 (G) showing iPSDMs expressing GFP–LC3B and infected with E2Crimson–Mtb WT (F) or ΔRD1 (G). Videos representative of thee biological replicates. Frames every 5 min for WT or 3 min for ΔRD1 infection. Arrows in F show bacteria that can be followed.

### Mtb infection induces RD1-dependent dynamic tubular autophagosomes in iPSDMs

Next, we used iPSDMs to investigate xenophagy in human macrophages after Mtb infection. When labelling autophagosomes with endogenous LC3B in Mtb-infected iPSDMs, we noticed that in addition to LC3B-positive membranes surrounding Mtb, large membranous compartments in the vicinity of Mtb were also labelled (Fig. 3A). This was observed after infection with Mtb WT but not with Mtb ΔRD1 (Fig. 3A). As shown before, ∼30% of Mtb were positive for LC3B at 2 h post infection (data not shown). Consistently, at 48 h of infection ∼40% of the total LC3B-positive Mtb displayed phenotypes similar to that shown in the WT example in Fig. 3A – which we named LC3 tubulovesicular structures (LC3-TVS) owing to their similarity to the structures observed during viral infection of epithelial cells (Gao et al., 2010) (Fig. 3B). To confirm this was not a phenomenon only observed in the iPSDMs, we examined hMDMs infected with Mtb WT or ΔRD1. Similar LC3-TVS were observed (Fig. 3C) at 2 h, although they were not observed at 24 h post infection (Fig. 3D). These observations suggested a local induction of LC3B-positive autophagosomes in the vicinity of Mtb in human macrophages. By using a transmission electron microscopy (TEM) approach, we similarly observed multiple membrane structures that were either containing or adjacent to Mtb WT (Fig. 3E). Since the dynamics of the autophagic targeting of Mtb in human macrophages has yet to be elucidated, we used the iPSDM model to monitor the spatiotemporal dynamics of GFP–LC3B during infection with Mtb. Strikingly, within 2 h of infection, Mtb WT triggered the local generation of large GFP–LC3B-positive membranous compartments – the LC3-TVS – that were often observed in proximity to, but not tightly associated with Mtb (Fig. 3F; Movie 2). These LC3-TVS appeared to be very stable, sometimes remaining for several hours. From these stable structures, thinner and transient tubules were also observed. Live-cell imaging of iPSDMs infected with Mtb ΔRD1 revealed no changes in LC3B localisation (Fig. 3G; Movie 3). Remarkably, we observed some bacteria that became transiently LC3B-positive before translocating away from the LC3B-positive membranes (Fig. 3F, Movie 2). Altogether, these results show that Mtb rapidly induces the formation of large and dynamic LC3B positive autophagosomes in an RD1-dependent manner.

### The formation of LC3-TVS occurs after phagosome membrane damage

**Fig. 4.**
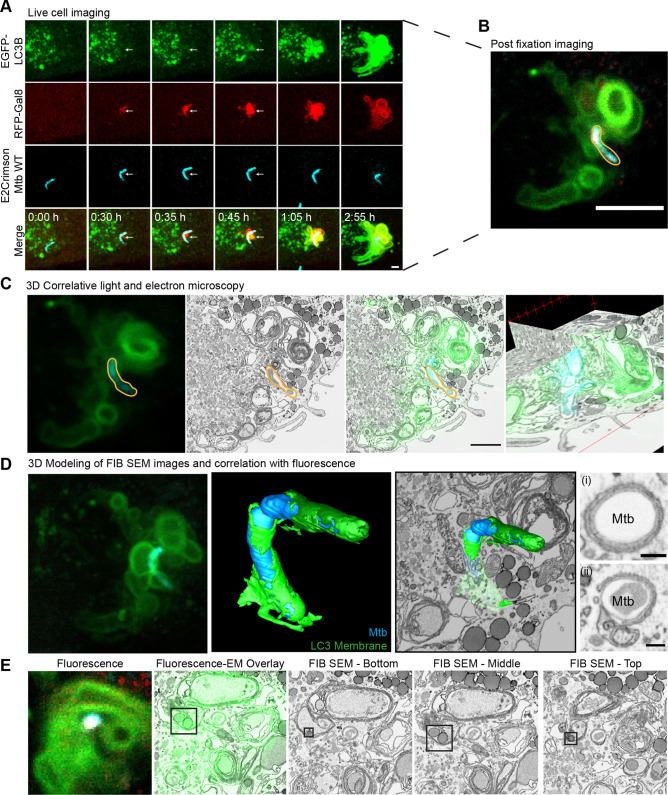
**Induction of LC3-TVS is preceded by Gal8 recognition of phagosome membrane damage.** iPSDMs expressing GFP–LC3B and RFP–Gal8 infected with E2Crimson Mtb WT were imaged live with frames every 5 min. (A) Maximum projection stills from live-cell imaging. Images were filtered using the Smooth function in ImageJ. Representative of two biological replicates. Arrows highlight the sequential recruitment of Gal8 and LC3B to the bacterium. Scale bar: 2 µm. (B) Single *Z*-slice from post-fixation confocal imaging of cell shown in A. Mtb outlined in orange. Scale bar: 5 µm. (C) 3D CLEM of post fixation *Z*-stack and FIB SEM of cell in A and B. Fluorescence and EM data were aligned using BigWarp. Mtb outlined in orange. Scale bar: 2 µm. (D) Segmentation of FIB SEM dataset showing the partial encasement of bacteria (cyan) in A and B in double-membrane structures (green) that are LC3-positive. Fluorescence image is a 3D projection of the post-fixation *Z-*stack. (i) shows a slice through the bacteria from the FIB SEM where the double membrane appears complete. (ii) shows a slice through the bacteria from the FIB SEM where the double membrane appears incomplete. Scale bar: 0.2 µm. (E) Correlation of fluorescence with FIB SEM section showing lipid droplets (black square) in a double-membrane LC3-positive compartment.

The ESX-1 dependency of the observed LC3-TVS in Fig. 3 led us to hypothesise that it was linked to phagosome membrane damage. To formally test this hypothesis, we carried out live-cell imaging of iPSDMs expressing GFP–LC3B and RFP–Galectin8 (Gal8; also known as LGALS8) (Movie 4). The membrane damage marker RFP–Gal8 was recruited to the Mtb-WT-containing phagosomes ∼15 min prior to GFP–LC3B and LC3-TVS were positive for both Gal8 and LC3B (Fig. 4A). Interestingly, the tubules extending from these structures were LC3B-positive but Gal8 negative. Post-fixation imaging of the cell in Fig. 4A revealed that many of the large LC3B-positive structures remained, although some were lost between live-cell imaging and fixation, suggesting that these events are very dynamic (Fig. 4B). To gain insights at the ultrastructural level, the cell in Movie 4 was analysed by three-dimensional correlative light and FIB SEM. This analysis identified the LC3-TVS to be mostly large, multi-membrane structures containing organelles and small vesicles (Fig. 4C). The bacteria were enwrapped by double membranes, reminiscent of endoplasmic reticulum (ER) structures, but likely to correspond to partially complete autophagosomes (Fig. 4D; Movie 5). Although the bacteria were surrounded by large autophagic structures, the FIB SEM analysis did not clearly reveal any direct contacts between the bacteria and the luminal contents of these autophagosomes (Fig. 4D; Movie 5). In some FIB SEM sections through the bacteria appeared to show a complete double membrane (Fig. 4Di), but in other planes it was possible to see distinct ends of the double membrane where it had not joined (Fig. 4Dii), highlighting the importance of a three-dimensional analysis. Interestingly, some of the autophagosomes contained lipid droplets and other small membranous structures, suggestive of lipophagy induction during Mtb infection (Fig. 4E; Movie 5).

### Mtb escapes from autophagosomes by triggering spatially segregated multi-membrane LC3B-positive structures

**Fig. 5.**
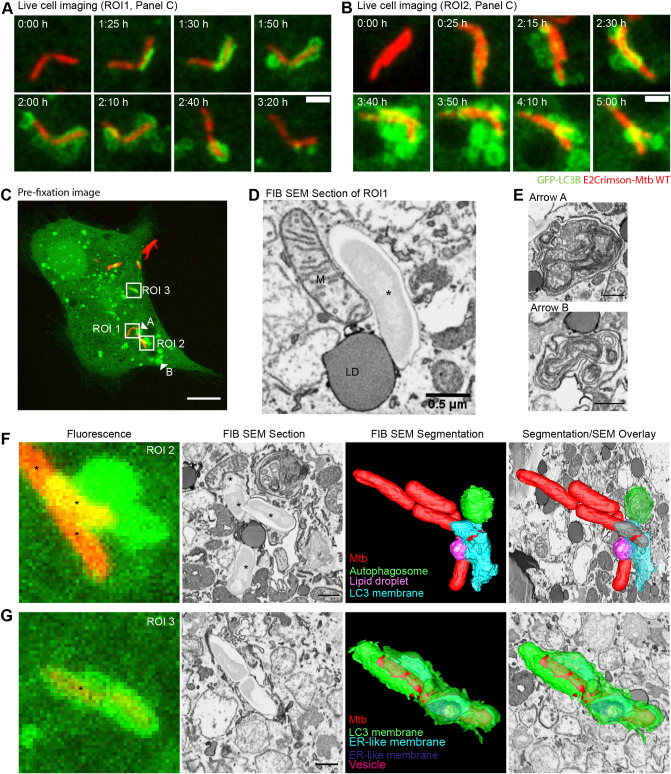
**Multi-membraned autophagosomes spatially segregate from cytosolic Mtb.** iPSDMs expressing GFP–LC3B (green) and infected with E2Crimson–Mtb WT (red) were followed by confocal live-cell imaging (A,B) with frames every 5 min. Images are maximum projection stills, filtered with a Gaussian blur, radius 0.8, showing two small clumps of bacteria from Movie 4. Time 0 for each bacterium represents frame of phagocytosis event. Scale bars: 2 µm. (C) Maximum projection of 15 *Z*-slice image of cell shown in A and B taken immediately prior to fixation. ROI1, 2 and 3 are shown in detail in D, F and G, respectively. Arrowheads A and B indicate LC3-positive membranes that were previously associated with Mtb and are shown in detail in E. Scale bar: 10 µm. (D) Section from FIB SEM showing one of the bacteria (*) in ROI1 in the cytosol in contact with a lipid droplet (LD) and a mitochondrion (M). (E) Section from FIB SEM showing LC3-positive membranes (indicated by arrowheads in C) that have shed from Mtb in ROI2. (F) Correlative light and FIB-SEM of ROI2 in C. Scale bar in FIB SEM section: 0.5 µm. (G) Correlative light and FIB-SEM of ROI3 in C. Scale bar in FIB SEM section: 0.5 µm.

To further characterise the Mtb-induced LC3-TVS at the ultrastructural level and determine the localisation of the bacteria after dissociating from the LC3-TVS, we performed correlative live-cell imaging and FIB SEM. Live-cell imaging showed the generation of local LC3B-positive structures, in addition to LC3B directly associated with Mtb WT (Fig. 5A,B; Movie 6). Immediately prior to fixation, a *Z*-stack of the cell of interest was captured, highlighting the localisation of LC3B-negative bacteria (ROI1), partially LC3B-positive bacteria (ROI2), entirely LC3B positive bacteria (ROI3) and LC3B puncta that had been shed from Mtb WT (arrowheads A and B) (Fig. 5C). Notably, mycobacteria that had been marked by LC3B, but were able to become spatially separated from these membranes were clearly localised in the cytosol by FIB SEM (Fig. 5D). When the LC3B-positive autophagosomes that became spatially separated from the bacteria were examined by correlative FIB SEM, they were revealed as complex multi-membrane compartments and often contained small, vesicle-like structures (Fig. 5E,F). Mtb WT bacilli partially enwrapped by an LC3B-positive membrane showed limiting membranes with invaginations and contained internal membranous structures (Fig. 5F; Movie 7). The FIB SEM-based three-dimensional analysis also revealed that mycobacteria in an LC3B-positive compartment were indeed almost entirely surrounded by a membrane that contained internal ER-like membrane sheets (Fig. 5G; Movie 8). Thus, LC3B-positive membranes and the bacilli tend to be spatially segregated with Mtb WT able to escape the autophagy machinery to reside in the cytosol.

## DISCUSSION

In the past decade, the use of iPSC-derived cells has revolutionised our molecular and cellular understanding of cell function, especially in the developmental biology and neuroscience fields (Hyman and Simons, 2011). The derivation of immune cells from iPSC is emerging as a novel strategy to investigate the cellular basis of both Mendelian and infection susceptibility/resistance in human disease (Zhang and Reilly, 2017). However, reports describing the use of iPSC to generate immune cells for the study of the cell biology of host–pathogen interactions are rather limited. Here, we show that iPSDMs represent a valuable tool for the generation of human macrophages enabling further study of human macrophage biology as well as dynamics in host cell–pathogen interactions in TB. iPSDMs have been used to study aspects of infectious diseases including *Chlamydia*, *Salmonella* and HIV (Hale et al., 2015; van Wilgenburg et al., 2013; Yeung et al., 2017). iPSDMs infected with the avirulent vaccine strain *M. bovis* BCG undergo apoptosis, caspase 3 activation and secrete TNF-α to similar levels to that observed with THP-1 macrophages (Hong et al., 2018).

Our analysis provides evidence for the use of iPSDMs as a relevant *in vitro* model that reflects well-described aspects of human primary macrophages. Firstly, flow cytometry characterisation shows that iPSDMs express surface markers of human macrophages, particularly after activation. Secondly, our RNA-seq analysis shows the expression of transcription factors associated with the macrophage lineage. Finally, at the functional level, iPSDMs are highly phagocytic, exhibit a typical macrophage morphology and actin cytoskeleton, and secrete pro-inflammatory cytokines after Mtb infection. The relatively low level of secretion of cytokines such as TNF-α and IL-6 is in keeping with findings showing these are also lower at basal levels in iPSDMs compared to hMDMs (Nenasheva et al., 2020).

Several aspects of our data support iPSDMa as a relevant system that reflects known outcomes of Mtb–human macrophage interactions *in vitro*. After infection, Mtb is able to replicate within iPSDMs in an RD1-dependent manner as shown for hMDMs (Lerner et al., 2017). In mouse macrophages, IFN-γ is well known to potently restrict Mtb replication, likely through the action of NO as well the autophagy pathway (Flesch and Kaufmann, 1987). In human macrophages, the situation is more controversial with several reports in hMDMs showing no, or very little effect of IFN-γ on Mtb replication (Douvas et al., 1985; Flynn and Chan, 2001; Lerner et al., 2017). In a recent study in iPSDMs, IFN-γ treatment was unable to restrict Mtb, as we also report here (Nenasheva et al., 2020). The gene expression analysis of infected iPSDMs identified the upregulation of key immune defence pathways, such as interferon signalling, and genes known to be dysregulated by Mtb infection. Our data is also consistent with previous transcriptional analysis of hMDM and human alveolar macrophages after Mtb infection (Papp et al., 2018).

Despite its critical role in the virulence of Mtb (Hsu et al., 2003; Pym et al., 2002), little is known about the pathways activated by the ESX-1 type VII secretion system in macrophages. How these pathways integrate the human macrophage response to Mtb ESX-1 activity is key to our understanding of TB pathogenesis. Our RNA-seq dataset is the first to identify the ESX-1-dependent response of human macrophages to Mtb infection, revealing novel pathways activated by this important virulence factor. Importantly, here we identified EIF2 signalling, PKR activation and activation of IRF by cytosolic PRRs as critical for the ESX-1-dependent response of human macrophages to Mtb infection. Notably, our gene expression analysis of infected iPSDMs identified the upregulation of interferon signalling. Over-representation of IFN genes has been seen in many RNA-seq analyses of blood from TB patients and is thought to be a good biomarker for disease progression (Berry et al., 2010; Moreira-Teixeira et al., 2018).

This work also validates the use of iPSDMs as a novel tool to study the cellular mechanisms of the human macrophage response to Mtb infection. In iPSDMs, Mtb WT efficiently replicates and localises predominantly in the cytosol by 48 h post infection. Conversely, Mtb ΔRD1 localises entirely in membrane bound compartments and shows restricted growth. This early ESX-1-dependent cytosolic access of Mtb has been reported in hMDMs and human monocyte-derived dendritic cells (Lerner et al., 2017; van der Wel et al., 2007), although the proportion of cytosolic bacteria observed in iPSDMs is significantly higher. However, our LTR experiments showing phagosome retention, leakage and recovery clearly highlight that the localisation of Mtb is very dynamic and suggest that competing processes of membrane damage and repair operate at the Mtb–phagosome interface. Moreover, our data in iPSDMs argue for cycles between both membrane-bound and cytosolic Mtb states, suggesting that Mtb faces fluctuating intracellular environments. These dynamic events are similar to those observed in mouse macrophages (Schnettger et al., 2017).

The spatiotemporal analysis of Mtb targeting to autophagic compartments in human macrophages uncovered strikingly complex membrane dynamics. This response seems to be rapid and relying on pre-existing cellular factors, given that at these early time points the transcriptional response was not different between Mtb WT- and Mtb ΔRD1-infected iPSDMs. Classically, Mtb is thought to be targeted to LC3B-positive compartments through ubiquitylation and potentially Gal8 recruitment (Franco et al., 2016; Manzanillo et al., 2013; Schnettger et al., 2017; Watson et al., 2012). However, our live-cell data show that LC3B is recruited to membranes that can spatially separate from the intracellular Mtb. Because these large membrane rearrangement events are dependent on ESX-1, firstly recognised by Gal8, and Mtb WT localises to the cytosol after these events, we conclude that they are triggered by membrane damage. This finding is reinforced by data in THP-1 macrophages showing Gal3 recruitment prior to autophagosome formation and tubulation, as well as spatial separation of these membranes from the bacteria (Beckwith et al., 2020). When compared to live imaging of GFP–LC3 in zebrafish larvae infected with *M. marinum*, our data shows differences (Hosseini et al., 2014). Hosseini et al. observed what appears to be a transient recruitment of LC3 to the bacteria, or LC3-positive vesicles contacting the bacteria but no tubules as we report here. These differences could be due to differences in either the pathogen or experimental model used. In the scenario observed in iPSDMs, it is likely that these damaged host membranes are targeted for autophagy-mediated degradation while the bacteria escape. Alternatively, this large membrane remodelling at the interface with the bacteria is an attempt by the host macrophage to repair the membrane damage inflicted by Mtb. Our data also provide evidence that some bacteria that become enclosed with LC3B-positive membranes can subsequently escape from them. How these damaged membranes are targeted to autophagy remains to be examined. Moreover, the fate of these tubules, as well as the source of membrane for their formation are currently unknown and require further study. Although non-canonical autophagy cannot be excluded, in iPSDMs, Mtb associated with LC3B only after phagocytosis, suggesting this is not LC3-associated phagocytosis. In this context, our data highlight that the situation is not as simple as Mtb localising either in single or double membrane compartments, and show that, in most cases, Mtb are surrounded by multiple membranes with very complex morphology. The ultrastructural characterisation of LC3B-positive membranes also revealed that these multi-lamellar structures appeared to retain some cytoplasmic content, and also contained small vesicular structures. Although, in iPSDMs, the formation of these structures was visualised by immunofluorescence to persist for over 48 h of infection, they were only observed at 2 h post infection in hMDMs.

The ultrastructure of the mycobacteria-containing compartment after membrane damage has previously been examined in other model systems. FIB SEM analysis of the damaged *M. marinum* phagosome and recruited autophagy machinery in *Dictyostelium discoideum* (López-Jiménez et al., 2018) showed a structure not dissimilar to that observed in Fig. 5. Additionally, multiple patterns of LC3 localisation to the *M. marinum* phagosome have been reported, including dots, patches and coats (Cardenal-Muñoz et al., 2017), with some of these structures resembling our live-cell imaging observations. Complex membrane structures with small, internal vesicles, reminiscent of those seen in our FIB SEM analysis, were also reported in the vicinity of Gal3-positive Mtb in THP1 cells (Beckwith et al., 2020).

While the role of autophagy in infection has mainly focused on direct targeting of bacteria to autophagosomes, there are reports of autophagy targeting membrane remnants and regulating inflammatory signalling. For example, *Shigella* vacuolar remnants can be cleared from epithelial cells by autophagic targeting through polyubiquitylation (Dupont et al., 2009). These ubiquitin-containing membrane remnants can recruit activated caspases to eliminate active inflammasomes to control inflammation (Mostowy, 2013). In a *M. marinum* model, polyubiquitin-containing dense membrane structures akin to those observed in our study were found in close proximity to the bacteria, and contained both host- and bacteria cell wall-derived components (Collins et al., 2009). Autophagic removal of ER segments undergoing ER stress in *Listeria*-infected macrophages is known to limit apoptotic cell death (Moretti et al., 2017).

As well as large, stable LC3B-positive membranes, we observed more transient LC3B-positive tubules. Similarly, LC3B-positive tubules have been observed in cells infected with *Staphylococcus aureus*. These *S. aureus* induced filaments are Rab7- and Rab1b-positive and require microtubules and kinesin 1 for their formation (López de Armentia et al., 2017). Interestingly, the pore forming toxin α-haemolysin is required for the formation of these tubules, further suggesting a link to membrane damage. Given that, in the context of both Mtb and *S. aureus*, a membrane-damaging component is required for the formation of these tubules, it is tempting to speculate that these membranes could be a mechanism for the removal of pore-inducing agents. For example, tubule formation and eventually fission will physically isolate toxins from endolysosomal membranes.

In summary, we introduce iPSDMs as a novel model to investigate human macrophage responses to Mtb that possess several advantages. iPSCs are amenable to genome editing by CRISPR/Cas9, allowing the production of genetically modified macrophages (Härtlova et al., 2018). It is feasible to knockout as well as knock-in fluorescent tags to proteins of interest, a technique which is not possible in primary human macrophages. Additionally, it is possible to derive iPSCs from people carrying a specific set of mutations and/or polymorphisms, making it possible to perform functional studies with macrophages from patients. This approach, combined with CRISPR-mediated correction of single mutations to generate isogenic lines will represent a powerful tool to investigate the role of macrophages in human disease.

## MATERIALS AND METHODS

### iPSDM culture and production

EIKA2 human iPSCs were sourced from Public Health England Culture Collections (catalogue number 66540072) and maintained in Vitronectin XF (StemCell Technologies) coated plates with E8 medium (ThermoFisher Scientific). Cells were authenticated by STR profiling upon receipt and are checked monthly for *Mycoplasma* contamination by PCR. Cells were passaged 1:6 once at 70% confluency using Versene (Gibco). Monocyte factories were set up following a previously reported protocol (van Wilgenburg et al., 2013). Briefly, a single cell suspension of iPSCs was produced with TryplE (Gibco) at 37°C for 5 min and resuspended in E8 plus 10 µM Y-27632 (Stem Cell Technologies) and seeded into AggreWell 800 plates (StemCell Technologies) with 4×10^6^ cells/well and centrifuged at 100 ***g*** for 3 min. The forming embryonic bodies (EBs) were fed daily with two 50% medium changes with E8 supplemented with 50 ng/ml hBMP4 (Peprotech), 50 ng/ml hVEGF (Peprotech) and 20 ng/ml hSCF (Peprotech) for 3 days. On day 4, the EBs were harvested by flushing out of the well with gentle pipetting and filtered through an inverted 40 µm cell strainer. EBs were seeded at 100–150 EBs per T175 or 250–300 per T225 flask in factory medium consisting of X-Vivo 15 (Lonza) supplemented with Glutamax (Gibco), 50 µM β-mercaptoethanol (Gibco), 100 ng/ml hM-CSF (Peprotech) and 25 ng/ml hIL-3 (Peprotech). These monocyte factories were fed weekly with factory medium for 5 weeks until plentiful monocytes were observed in the supernatant. Up to 50% of the supernatant was harvested weekly and factories fed with 10–20 ml factory medium. The supernatant was centrifuged at 300 ***g*** for 5 min and cells resuspended in X-Vivo 15 supplemented with Glutamax, 50 µM β-mercaptoethanol and 100 ng/ml hM-CSF and plated at 4×10^6^ cells per 10 cm petri dish to differentiate over 7 days. On day 4, a 50% medium change was performed. To detach cells, iPSDM plates were washed once with PBS then incubated with Versene for 15 min at 37°C and 5% CO_2_ before diluting 1:3 with PBS and gently scraping. Macrophages were centrifuged at 300 ***g*** and plated for experiments in X-Vivo 15 plus Glutamax and 50 µM β-mercaptoethanol.

### Human monocyte-derived macrophage production

CD14-positive monocytes were purified from Leukocyte cones (NHS Blood and Transplant) as described previously (Lerner et al., 2017) and differentiated to macrophages in the presence of 50 ng/ml M-CSF in RPMI plus 10% FCS.

### Mtb infection

Mtb H37Rv WT and Mtb H37Rv ΔRD1 were kindly provided by Prof. Douglas Young (The Francis Crick Institute, UK) and Dr Suzie Hingley-Wilson (University of Surrey, UK). Fluorescent Mtb strains were generated as previously reported (Lerner et al., 2016). E2Crimson Mtb was generated by transformation with pTEC19 (Addgene 30178, deposited by Prof. Lalita Ramakrishnan). Strains were verified by sequencing and tested for PDIM positivity by thin layer chromatography of lipid extracts from Mtb cultures. Mtb strains were cultured in Middlebrook 7H9 supplemented with 0.2% glycerol, 0.05% Tween-80 and 10% albumin dextrose catalase (ADC). For macrophage infections, Mtb was grown to OD_600_∼0.8 then centrifuged at 2000 ***g*** for 5 min. The pellet was washed twice with PBS, then the pellet was shaken with 2.5–3.5 mm glass beads for 1 min to produce a single-cell suspension. The bacteria were resuspended in 10 ml cell culture medium and centrifuged at 300 ***g*** for 5 min for 5 min to remove clumps. The OD_600_ was determined and bacteria diluted to an appropriate OD for the required multiplicity of infection (MOI) – assuming OD_600_=1 equates to 10^8^ bacteria/ml – before adding to cells in a minimal volume. After 2 h, the inoculum was aspirated, cells washed twice with PBS and fresh culture medium added. Cells were then incubated for appropriate time points before collecting for analysis as described in the sections below. For all infections an MOI of 2 was used, except for replication experiments where an MOI of 1 was used.

### Immunofluorescence

Infected cells on No. 1.5 glass coverslips were fixed in 4% paraformaldehyde (PFA) overnight at 4°C. Samples were quenched in 50 mM NH_4_Cl for 10 min, permeabilised in PBS with 0.05% Saponin and 0.1% BSA for 10 min and blocked in PBS with 0.1% BSA for 5 min. Primary antibody diluted in permeabilisation solution was added for 1 h at room temperature. Coverslips were washed three times with PBS then incubated with secondary antibody for 1 h at room temperature. After staining cells were washed twice in PBS, incubated with DAPI for 10 min, washed twice more and mounted on glass slides using DAKO mounting medium (DAKO). Antibodies used were anti-LC3B antibody (MBL PM036, 1:100) and anti-rabbit-IgG conjugated to Alexa Fluor 488 (Life technologies A11034, 1:800). Samples were imaged on a laser-scanning confocal microscope (SP5 or SP8, Leica Biosystems) with a 63×/1.4NA oil immersion objective. Alternatively, cells were infected in a 96-well glass bottom ViewPlates (Perkin Elmer) and imaged on an Opera Phenix (Perkin Elmer) with 40× water objectives.

### Western blotting

Samples were lysed on ice in RIPA buffer (Millipore) supplemented with protease inhibitor cocktail (Sigma-Aldrich) then boiled in LDS sample buffer and NuPage reducing agent (Invitrogen) at 95°C for 10 min. Samples were loaded onto 4-12% bis-Tris SDS-PAGE (Invitrogen) gels for electrophoresis and then transferred to PVDF membranes using an iBlot2, program P0. Membranes were blocked in 5% skimmed milk powder in PBS plus 0.05% Tween20 (PBS-T) for 1 h at room temperature then incubated with primary antibody overnight at 4°C. Membranes were washed in PBS-T and incubated with HRP-conjugated secondary antibodies for 1 h at room temperature. Membranes were developed with enhanced chemiluminescence reagent (BioRad) and imaged on an Amersham GE Imager. Antibodies used were anti-IDO (New England Biolabs #12006 1:1000), anti-iNOS (Novus Biologicals #NBP1-67618 1:250), anti-actin conjugated to HRP (Cell Signaling Technologies #13E5 1:5000) and anti-rabbit-IgG conjugated to HRP (Promega W4011 1:10,000) antibodies.

### Nitric oxide detection

200 µl of supernatant was collected from iPSDMs treated with 500 ng/ml LPS, 20 ng/ml IFN-γ or both in combination overnight. Samples were then analysed for levels of nitrite and nitrate using a Nitric Oxide Assay Kit (ThermoFisher Scientific, EMSNO) following the manufacturer's instructions.

### iPSDM electroporation

Plasmid DNA was electroporated into iPSDMs using the Neon system (Invitrogen). iPSDM were resuspended at 2×10^6^ cells in 100 µl buffer R and 1 µg plasmid DNA per electroporation added. 10 µl of cell/DNA mix was aspirated into a Neon pipette and electroporated in electroporation buffer ‘E’ at 1700 V for 30 ms with 1 pulse. Cells were plated in gridded CLEM dishes (Mattek) and allowed to adhere before additional culture medium was added.

### Live-cell imaging

After addition of bacteria, Mattek gridded dishes were sealed with parafilm, loaded into a holder and imaged on a laser scanning confocal microscope (SP5, Leica Biosystems) at 37°C/5% CO_2_ using a custom-made environmental chamber from EMBL. Samples were imaged using a 63×/1.4NA oil immersion objective.

For live imaging of Lysotracker, cells were stained for 15 min with 50 nM Lysotracker Green DND-26 (ThermoFisher Scientific) at 37°C/5%CO_2_ before removal of media and addition of Mtb and immediate transfer to the microscope and imaging as described above.

### Image analysis

To quantify Lysotracker association, bacteria of interest were duplicated from the field of view and a mask of the bacteria and a ring of pixels around the bacteria was generated. To do this the bacteria-containing channel was thresholded using manually determined range and a single ‘Dilate’ command was applied to expand the mask by 1 pixel in ImageJ. This mask was then used to measure the mean fluorescence intensity of pixels in the green (Lysotracker) channel in the area defined by the mask using ‘Measure’ as previously reported (Schnettger and Gutierrez, 2017).

To quantify Mtb replication images were acquired on an Opera Phenix (Perkin Elmer) using a 40× objective with at least 15 fields of view per well with a 10% overlap between adjacent fields, and three wells per condition per experiment and analysed in Harmony (Perkin Elmer Inc.). Fields were stitched together to create a global image with dynamic binning, and cells were segmented based on DAPI, excluding any cells touching the edge of the imaged area. Bacteria were detected using the ‘Find Spots’ building block of Harmony. The total bacterial area in each cell was then determined. Data was exported and analysed in RStudio to calculate the mean Mtb area per cell for each condition at each timepoint, with all three wells pooled. This was used to calculate the fold change, relative to time 0, in mean Mtb area per cell.

### Flow cytometry

Cells were stimulated with 20 ng/ml IFN-γ plus 500 ng/ml LPS overnight as indicated in figure legend. Cells were collected and incubated in PBS plus 0.1% BSA and 5 µl FC block per million cells for 20 min. 50 µl of cells were then incubated with 50 µl antibody cocktail diluted in PBS and 0.1% BSA for 20 min on ice in the dark. Cells were washed in 2 ml PBS and fixed in 2% PFA prior to analysis. Cells were analysed on an LSRII flow cytometer. Antibodies were purchased from BD Biosciences and are detailed in Table S1. Flow cytometry data was analysed and plotted in FlowJo (BD Biosciences).

### Transmission electron microscopy

To prepare samples, cells were washed in PBS and then fixed in 2.5% glutaraldehyde (GA) in 200 mM HEPES pH 7.4 for 30 min at room temperature then overnight at 4°C. After several washes in 200 mM HEPES buffer, samples were processed in a Pelco Biowave Pro (Ted Pella, USA) with use of microwave energy and vacuum. Briefly, samples were fixed and stained using a reduced osmium, thiocarbohydrazine, osmium (ROTO)/en bloc lead aspartate protocol (Lerner et al., 2017). Samples destined for stereological analysis were dehydrated using an ethanol series of 50, 75, 90 and 100% then lifted from the tissue culture plastic with propylene oxide, washed four times in dry acetone and transferred to 1.5 ml microcentrifuge tubes. Samples were infiltrated with a dilution series of 50, 75 or 100% (v/v) of Ultra Bed Low Viscosity Epoxy (EMS) resin to acetone mix and centrifuged at 600 ***g*** between changes. Finally, samples were cured for a minimum of 48 h at 60°C before trimming and sectioning.

For sectioning and imaging, ultrathin sections (∼50 nm) were cut with an EM UC7 Ultramicrotome (Leica Microsystems) using an oscillating ultrasonic 35° diamond Knife (DiaTOME) at a cutting speed of 0.6 mm/sec, a frequency set by automatic mode and, a voltage of 6.0 volts. Images were acquired using a 120 kV Tecnai G2 Spirit BioTwin (FEI company) with an Orius CCD camera (Gatan Inc.)

### Scanning electron microscopy

iPSDMs were treated with an inoculum of Mtb WT at an MOI of 10 for 20 min on 13 mm glass coverslips. Samples where then fixed with a mixture of 4% PFA, 1.25% GA, 0.04 M sucrose in 200 mM HEPES pH 7.4 overnight at 4°C. A Biowave Pro was used to process the samples as follows. Samples were washed twice in HEPES at 250 W for 40 s and post-fixed in 2% osmium tetroxide (Taab). Samples were washed in 200 mM HEPES then dehydrated using a step-wise ethanol series of 50, 75, 90 and 100% at 250 W for 40 s per step. Samples were critical point dried using an EM CPD300 (Leica Microsystems, Germany) and acetone as the solvent. The samples were coated with 2 nm of gold before imaging on a Quanta SEM (FEI, United States).

### Stereology analysis

To extract quantitative information about a three-dimensional material from measurements made on two-dimensional sections of Mtb-infected iPSDM, we performed stereology. Mtb-infected iPSDMs were washed once with 200 mM HEPES then fixed for 30 min in 2.5% GA at room temperature, then overnight at 4°C. The following day the fixative was exchanged for 1% GA. Cells were then pelleted at 600 ***g*** after detachment from tissue culture plastic prior to embedding. At least 24 different infected cells per sample were imaged at 3900× magnification by systematic and random sampling. Cross points of the stereological test grid over bacteria were counted with regard to the subcellular localisation of bacteria, which was determined from images taken at minimum magnification of 16,000×. Percentages were calculated from total counts per sample. The following criteria were followed for the assessment of subcellular membrane involvement: (1) single surrounding membrane – bacteria were, at least partially, tightly lined by a phospholipid bilayer, representing the phagosomal membrane; (2) cytosolic – bacteria were surrounded by ribosomes, representing the cytoplasm with no indication of the phagosomal membrane; (3) multiple surrounding membranes – bacteria were enveloped by double or multiple membrane structures.

### Correlative live-cell fluorescence and FIB SEM

Sample preparation was performed as follows. iPSDMs expressing GFP–LC3B (Addgene #21073) and/or RFP-Gal8 (subcloned in house from Addgene #68805 into pmRFP-C1 using BglII and KpnI) were imaged live as described above before a final high-resolution *Z*-stack was acquired. Samples were fixed in 4% PFA overnight at 4°C before high-resolution *Z*-stacks of the fixed cells were acquired. Cells were then fixed in 2.5% GA for 15 min at room temperature before being processed for EM as described above, with the exception of using Durcupan resin. Cells were seeded onto 35 mm gridded Mattek dishes with a No. 1.5 coverslip (MatTek Corporation), infected and imaged as described, then fixed in 4% PFA in PBS overnight at 4°C. Cells were then fixed in 1% GA in 200 mM HEPES pH7.4 for 30 min at room temperature. Samples were processed in a Pelco Biowave Pro as described above except only being washed twice with dry acetone. In the next steps, samples were infiltrated with a dilution series of 50, 75 and 100% of Durcupan™ resin (Sigma-Aldrich) to acetone mix. Finally, samples were cured for a minimum of 48 h at 60°C.

FIB SEM data was collected using a Crossbeam 540 FIB SEM with Atlas 5 for three-dimensional tomography acquisition (Zeiss, Cambridge). The grid square containing the cell of interest was trimmed from the resin block, mounted on a standard 12.7 mm SEM stub using silver paint, and coated with a 5 nm layer of platinum. The specific cell of interest was relocated in the SEM by imaging through the platinum coating at an accelerating voltage of 10 kV and correlating to previously acquired fluorescence microscopy images. After preparation for milling and tracking, electron micrographs were acquired at 5 nm isotropic resolution throughout each region of interest, using a 9 or 10 µs dwell time. During acquisition, the SEM was operated at an accelerating voltage of 1.5 kV with 1 nA current. The EsB detector was used with a grid voltage of 1200 V. Ion beam milling was performed at an accelerating voltage of 30 kV and current of 700 pA.

After cropping to each region of interest, the datasets were aligned (register virtual stack slices, translation, default settings; Fiji), and batch processed to suppress noise, and enhance sharpness and contrast [gaussian blur (0.8 pixel radius), smart sharpen (60%, 10 pixel radius), smart sharpen (100%, 2 pixel radius), medium contrast curve; Adobe Photoshop CC 2015.5]. For viewing, and to mitigate any misalignment with respect to the light microscopy orientation that was introduced during specimen mounting, each dataset was rotated and resliced in *XY* and *YZ* planes to flatten the volume with respect to the coverslip surface. The volume was then resliced in the *XZ* plane, perpendicular to the coverslip surface, to enable direct correlation of light and electron microscopy images.

3D alignment of the fluorescence and FIB SEM images was performed using BigWarp. Fluorescence and FIB SEM files were converted to tiff files and liner adjustments made to brightness and contrast using Fiji (version 2.0.0-rc-69/1.52p). The BigDataViewer and BigWarp plugin (Pietzsch et al., 2015) was used to align the two datasets. The FIB-SEM stack was set as ‘target’ and the light microscopy stack as ‘moving’ dataset. In landmark mode, a minimum of ten fiducials were placed in the first instance, across the six-axis (*x*, *y*, *z*, pitch, yaw, roll) for the initial alignment. Further points were chosen to increase the accuracy using a thin-plate spline method. 2D CLEM of single slices from the fluorescence and FIB SEM was performed in Icy using the ecCLEM plugin with the bacteria and clearly defined LC3 positive structures as landmarks.

### FIB-SEM segmentation

Datasets were imported into IMOD (Kremer et al., 1996) and manually segmented. The cytosol-facing membranes were outlined. Where gaps existed in membranes or there was ambiguity as to the presence or location of a membrane, they were introduced into the model and left as holes. Where the sample was torn during coverslip detachment and this affected sample preservation, a hole was left.

### RNA sequencing sample preparation

10^6^ EIKA2 iPSDMs were seeded in six-well plates and infected at an MOI of 2 for 2 h. Extracellular bacteria were removed with two PBS washes, and cells were then incubated in macrophage medium for 2 or 48 h. At the respective time point samples were incubated in 1 ml TRIzol at room temperature for 5 min. Samples were stored at −80°C until ready for library preparation. Samples were collected from three independent biological replicates.

### RNA sequencing library preparation

RNA libraries were constructed using TruSeq Stranded Total RNA Library Prep Gold kit (Illumina) where ribosomal and mitochondrial RNA were depleted by RiboZero beads according to manufacturer's protocol. The final libraries were assessed using TapeStation 2200 System (Agilent). All libraries were sequenced on Illumina Hi-Seq 4000 instrument with SR75 reactions.

### RNA-Seq data analysis

The quality of the sequencing fastq files was analysed using FastQC (v0.11.5) and low-quality samples were excluded from further differential gene expression analysis. Sequence reads were adapter- and quality- trimmed using Trimmomatic (v0.36) before aligning to the human genome (Ensembl GRCh38 build 88) using STAR aligner (v2.5.2a). Gene expression was quantified using RSEM (v.1.2.29) and differential gene expression analysis was performed using DESeq2 (v1.20.0) with default parameters. DESeq2 uses a generalised linear model to estimate log2 fold change (log2FC) between comparison groups and the Benjamini-Hochberg false discovery rate was applied for multiple testing corrections, resulting in an adjusted *P*-value (padj) for each gene per comparison. Functional canonical pathways and disease networks associated with the differentially expressed genes were identified using Ingenuity IPA (QIAGEN).

### Data repository

All RNA-Seq data has been deposited to Gene Expression Omnibus with accession number GSE132283.

### Luminex

A total of 200 µl of supernatant from infected cells in a 96-well plate was collected and filtered twice through 0.2 µm PVDF membranes and stored at −80°C. Cytokine levels were analysed using a 20plex human inflammation panel from Invitrogen (EPX200-12185-901) according to the manufacturer's instructions. Samples were analysed on a Bio-Plex 200 (BioRad).

### Statistical analysis

Statistical analysis was performed using GraphPad Prism 8.4 for Windows, (GraphPad Software, La Jolla California) or R Studio. Analyses performed are outlined in the figure legends.

### Supplemental material

Refer to Fig. S1 for iPSDM set up and characterisation, and Fig. S2 for further RNA-seq data. See Movies 1-8 that correspond to stills shown in Figs 1 and 3–5.

## Supplementary Material

Supplementary information
