# Supplementary material for: M. tuberculosis infection of human iPSC-derived macrophages reveals complex membrane dynamics during xenophagy evasion
Source: J Cell Sci. 2020 Nov 25;134(5):jcs252973. doi: 10.1242/jcs.252973 (PMC7710011; doi:10.1242/jcs.252973)
Supplement: Supplementary information [file joces-134-252973-s1.pdf]

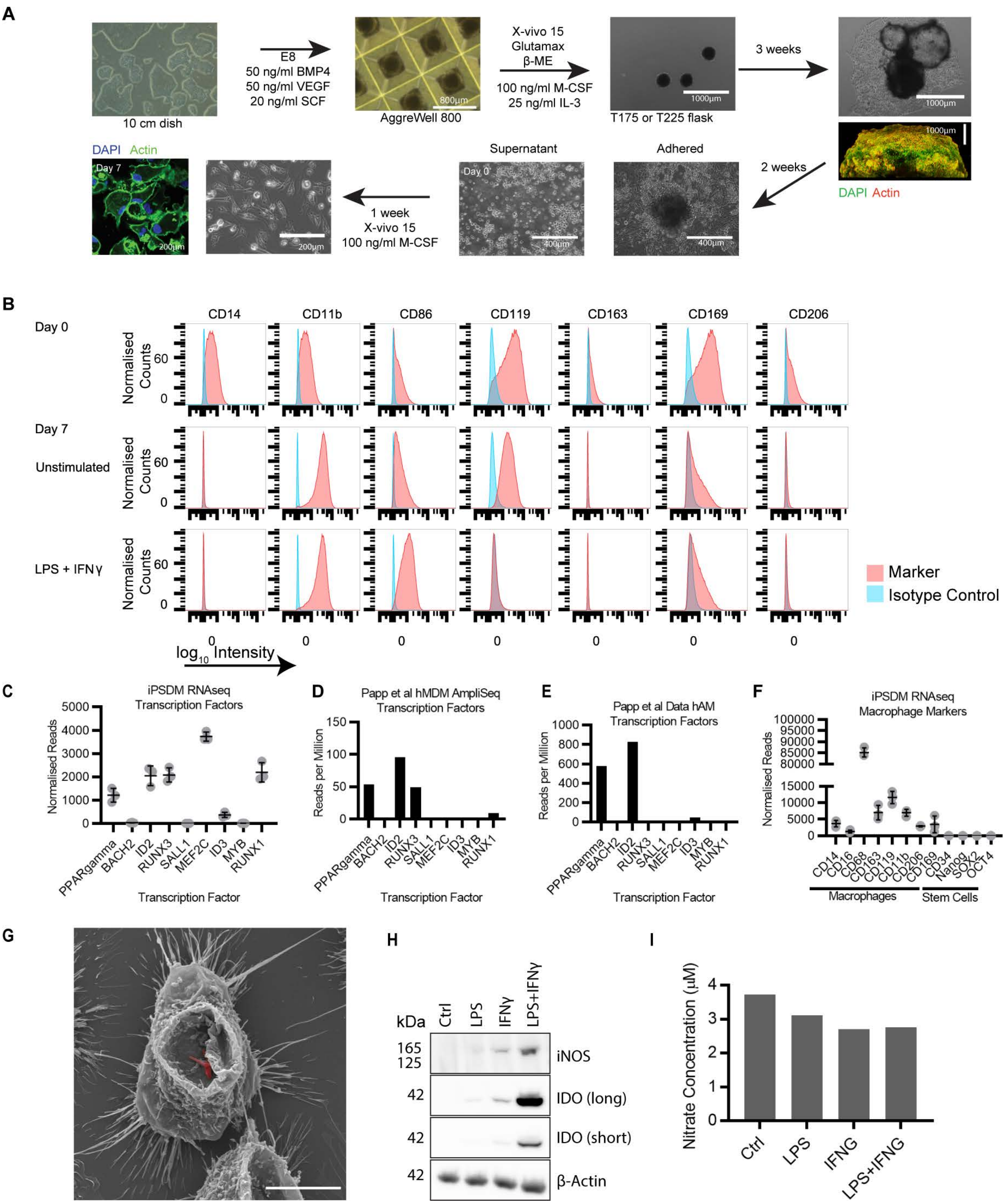

Journal of Cell Science • Supplementary information

Figure S1. The model of iPSDM to study Mtb-macrophage interactions. (A) Derivation protocol of iPSDM from iPSC via embryonic bodies. Light sheet microscopy shows the 3D nature of the EB, scale bar 300 µm. Actin staining of macrophages shows classical cortical actin staining. (B) Flow cytometry characterisation of iPS monocytes and macrophages, including the response of iPSDM to LPS + IFN-γ stimulation. Blue – Isotype control, pink – Marker. Representative of two biological replicates. (C) Normalised reads for several macrophage specific transcription factors from 2 h uninfected control condition from RNAseq experiment described in Figures 2 & S2. Data are mean ± SD from 3 biological replicates, each replicate is shown as a point on the graph. (D&E) Data from Papp *et al* 2018 hMDM (D) or human alveolar macrophage (E) AmpliSeq datasets for the same transcription factors in (C). (F) Normalised reads for several macrophage and stem cell specific markers from the 2 h uninfected samples from the RNAseq experiment. (G) Scanning electron micrograph of an iPSDM phagocytosing Mtb (red). Scale bar 5 µm. (H) Western blot of iNOS and IDO expression in iPSDM following exposure to 500 ng/ml LPS and/or 20 ng/ml IFN-γ. Data from 1 biological replicate. (I) Detection of nitrate in supernatant of iPSDM stimulated with LPS and IFN-γ for 24 h. Data from 1 biological replicate.

Journal of Cell Science • Supplementary information

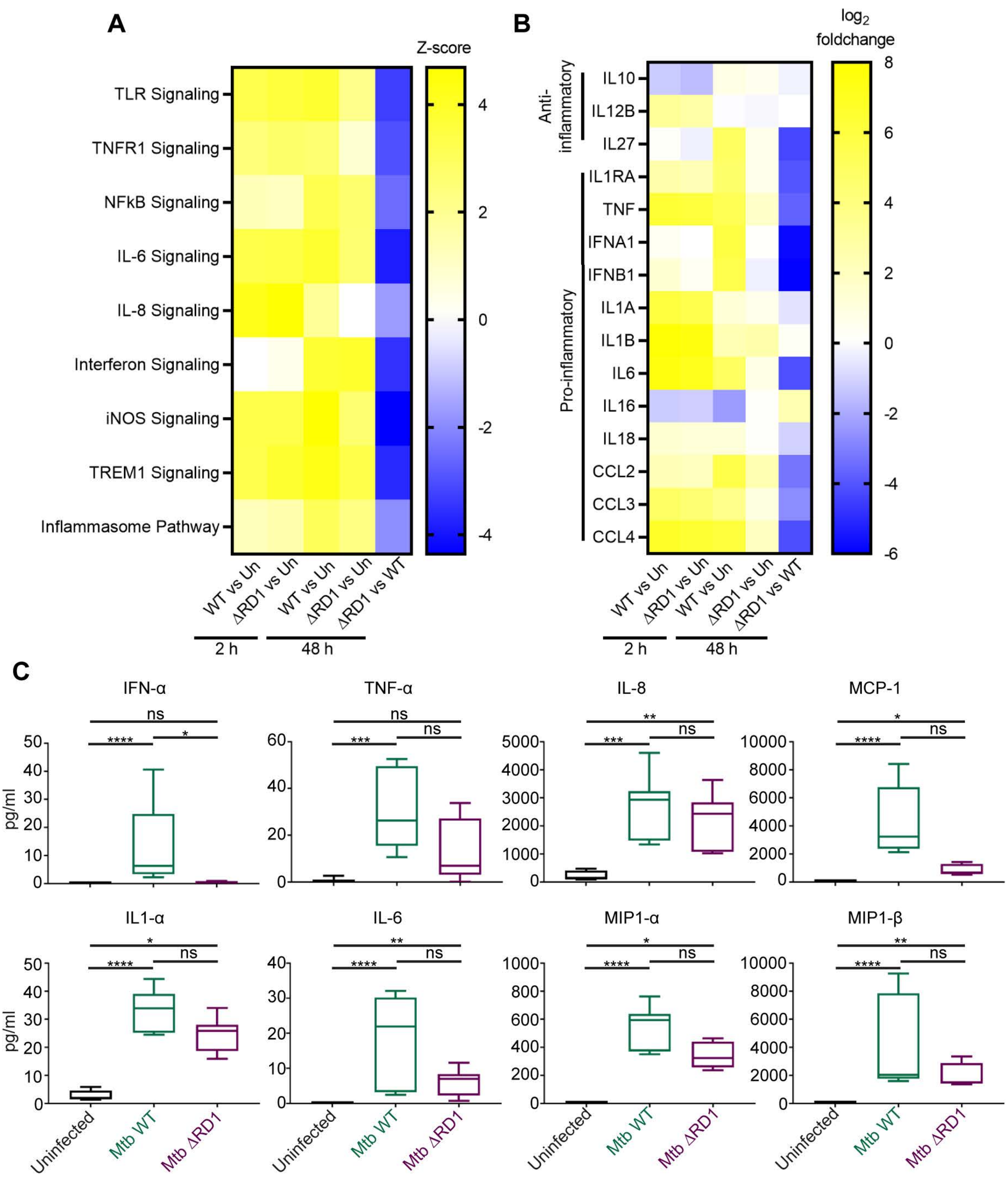

Figure S2. RD1 dependent inflammatory responses in iPSDM. (A) Heat map showing pathway activation, measured by Z score, of selected immune signalling pathways. (B) Heat map showing log$_2$foldchange of selected cytokines and chemokines from RNA-seq data presented previously. (C) Supernatants from uninfected or 48 h Mtb WT or ΔRD1 infected iPSDM were analysed by Luminex for cytokine secretion. Selected cytokines are shown. Data was analysed with Dunn's multiple comparisons test, ns not significant, * p<0.05, ** p<0.01, *** p<0.001, ****

p<0.0001. Data are medians, with whiskers showing minimum and maximum from 3 biological replicates each with 3 technical replicates.

Table S1. Antibodies used for flow cytometry. Source: BD Biosciences

**Antibody**	Clone	Volume used (µl)
**CD14-Alexa647**	MΦP9	5
**CD119-PE**	GIR-208	5
**CD86-BV421**	2331	5
**CD11b-APC**	ICRF44	20
**CD163-FITC**	GHI/61	5
**CD169-PE**	7-239	5
**CD206-BV421**	19.2	5
**IgG1κ-PE**	-	20
**IgG1κ-FITC**	-	5
**IgG1κ-APC**	-	20
**IgG1κ-BV421**	-	5
**IgG2bκ-Alexa647**	-	5

Journal of Cell Science • Supplementary information

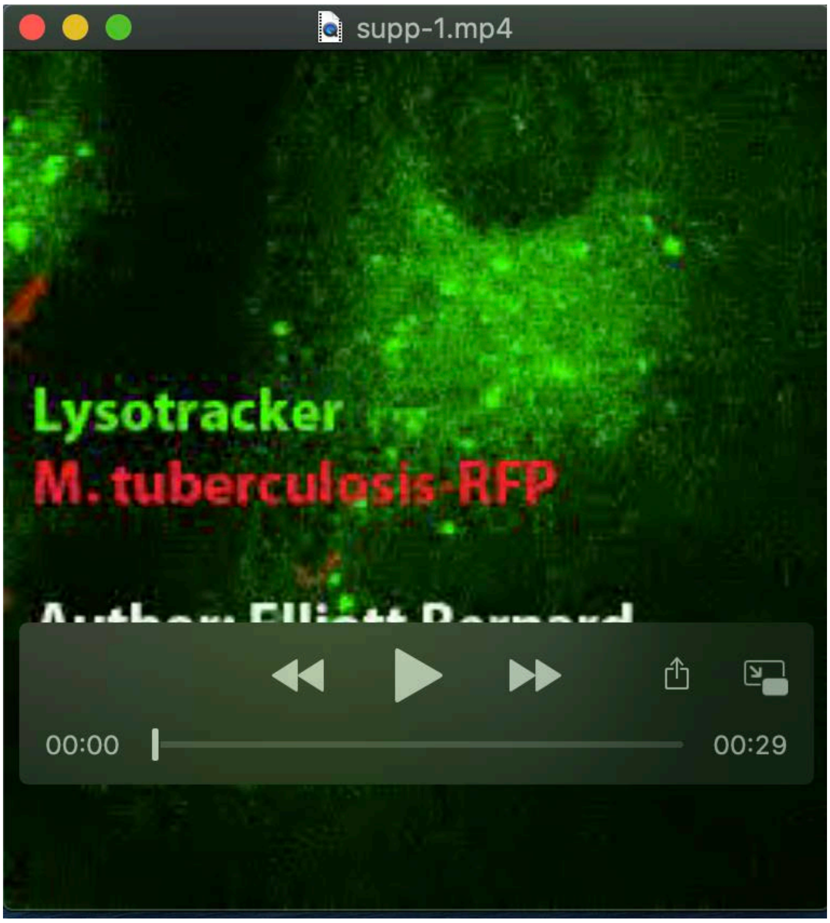

Movie 1. LTR positive Mtb phagosomes become leaky suggesting membrane permeabilisation. Maximum projection of time-lapse confocal microscopy images showing E2Crimson-Mtb WT (red) infected iPSDM stained with Lysotracker Green (green). Frames were every 5 min, with 6 Z-slices. Time 0 represents time of phagocytosis. Images were filtered with a Gaussian blur, radius 0.8. Presented at 7 frames per second. Stills from this video are presented in figure 1.

Journal of Cell Science • Supplementary information

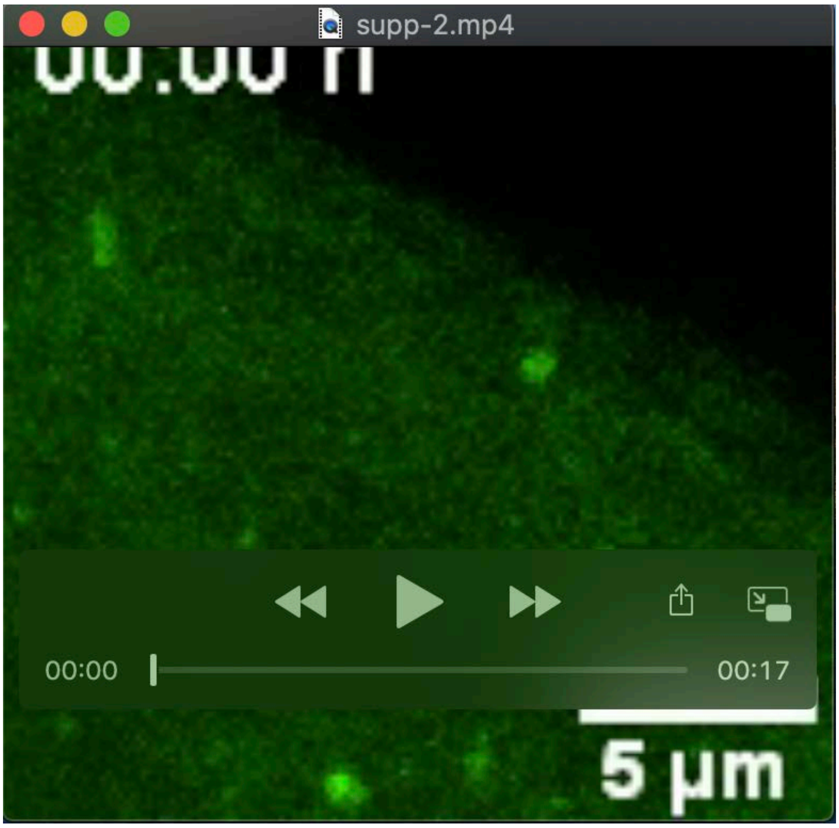

Movie 2. Mtb WT induces LC3-TVS formation. iPSDM expressing GFP-LC3B (green) were infected with E2Crimson Mtb WT (red) and followed by live cell confocal microscopy with frames every 5 min. Time 0 is the frame prior to phagocytosis. Movie is a maximum projection of 7 Z slices. Scale bar 5 µm, arrow indicates bacterium of interest. Presented at 10 frames per second. Stills from this movie are presented in figure 3.

Journal of Cell Science • Supplementary information

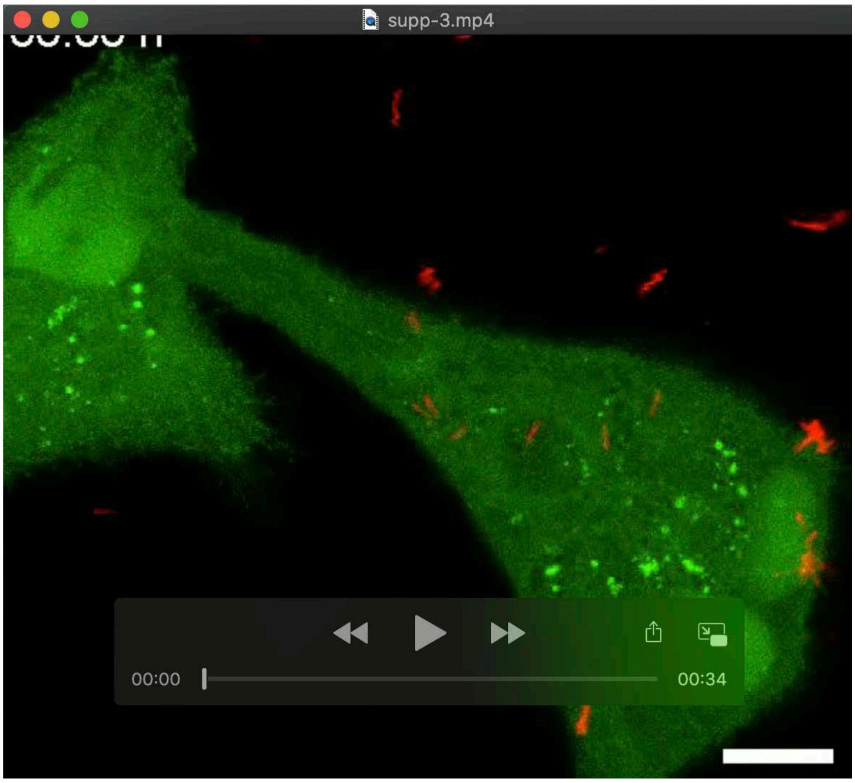

Movie 3. Mtb ΔRD1 fails to induce LC3B recruitment to the bacteria or other membranes in the vicinity of the phagosome. iPSDM expressing GFP-LC3B (green) were infected with E2Crimson Mtb ΔRD1 (red) and followed by live cell confocal microscopy with frames every 5 min. Movie is a maximum projection of 8 Z slices. Scale bar 10 μm. Presented at 10 frames per second. Stills from this video are presented in figure 3.

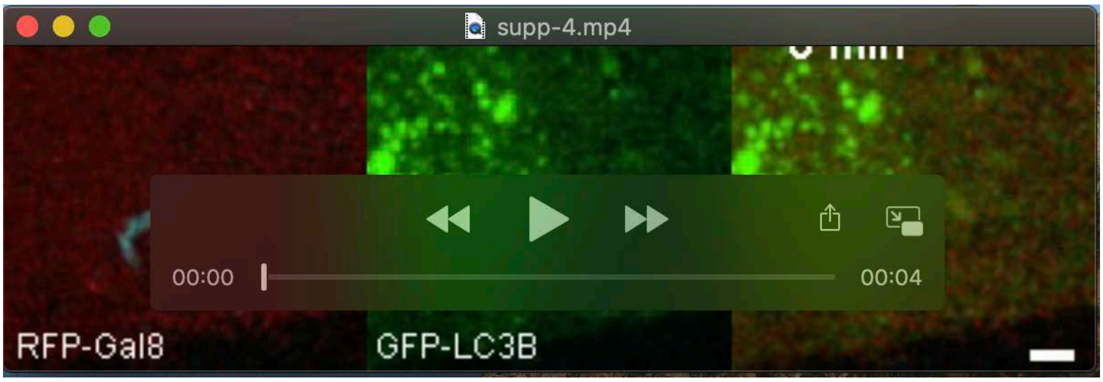

Movie 4. Induction of LC3-TVS is preceded by Galectin 8 recognition iPSDM expressing GFP-LC3B (green) and RFP-Gal8 (red) were infected with E2Crimson Mtb ΔRD1 (cyan) and followed by live cell confocal microscopy with frames every 5 min. Movie is a maximum projection of 7 Z slices. Scale bar 10 µm. Presented at 5 frames per second. Stills from this video are presented in figure 4.

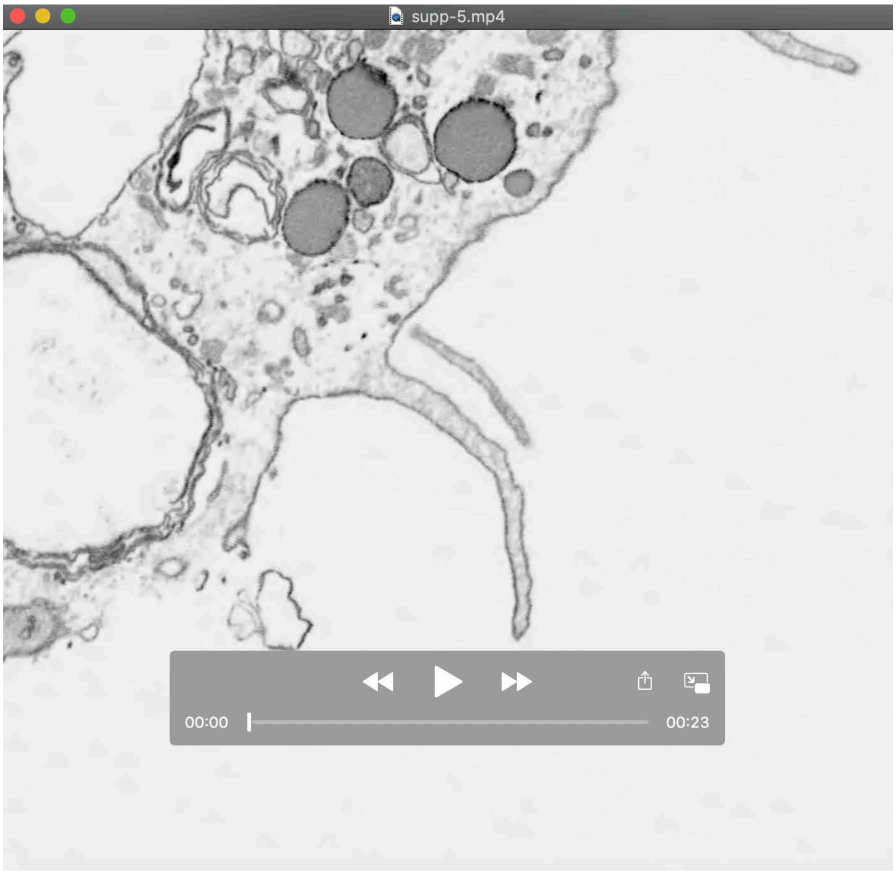

Movie 5. FIB SEM reconstruction of Mtb WT partially enwrapped in an LC3 positive membrane. 3D reconstruction of FIB SEM images obtained for cell shown in figure 2. The limiting membrane of the bacteria (cyan) and LC3 positive membranes surrounding the bacteria (green) were manually segmented. Where the presence of a membrane was ambiguous a gap was left. Presented at 10 frames per second. Stills from this video are presented in figure 4.

Journal of Cell Science • Supplementary information

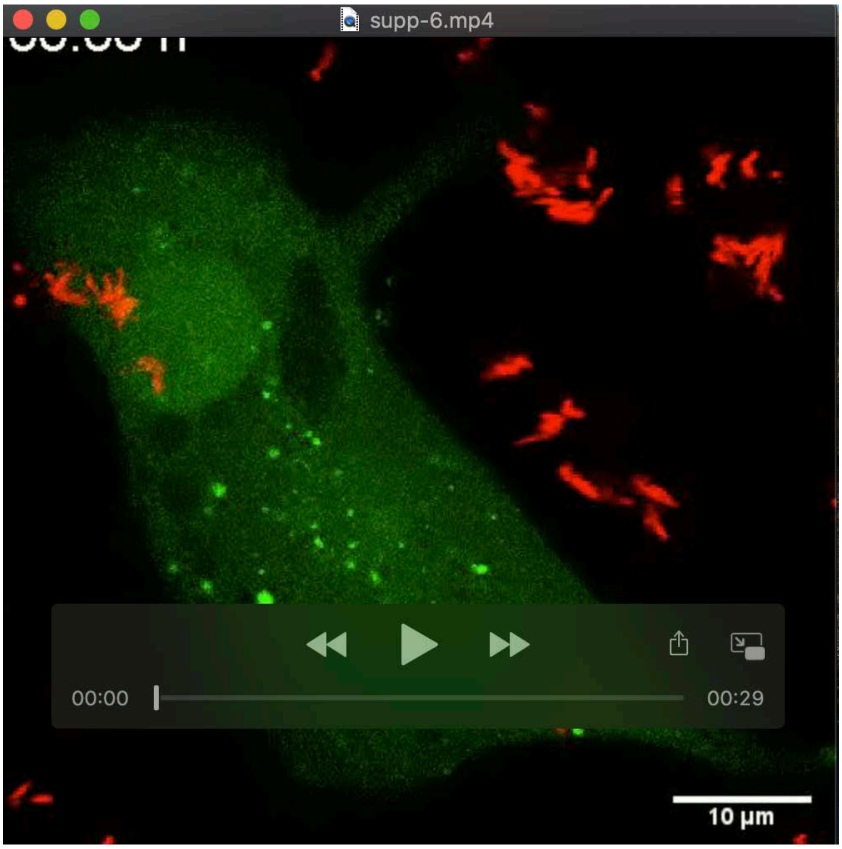

Movie 6. Mtb WT induced LC3B positive autophagosomes are spatially separated from the bacteria. iPSDM expressing GFP-LC3B (green) were infected with E2Crimson Mtb WT (red) and followed by live cell confocal microscopy with frames every 5 min. Movie is a maximum projection of 7 Z slices. Scale bar 10 µm. Presented at 10 frames per second. Stills from this video are presented in figure 5.

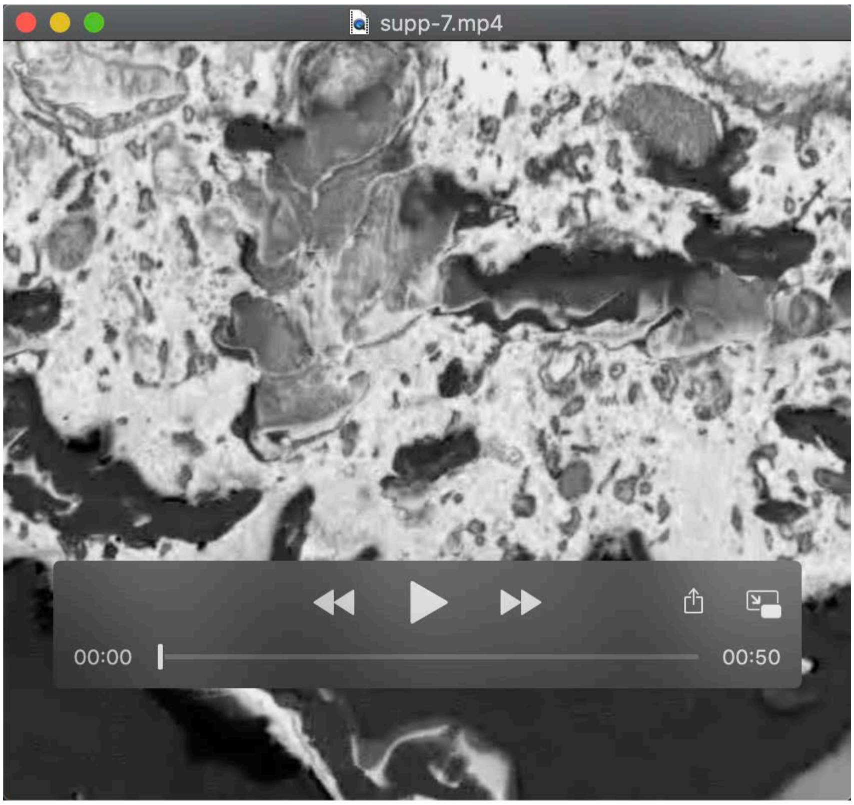

Movie 7. FIB SEM reconstruction of Mtb WT partially enwrapped in an LC3B positive membrane. 3D reconstruction of FIB SEM images obtained for ROI2 4. The limiting membrane of the bacteria (red), LC3 positive vacuole (green), LC3 positive membrane around Mtb (cyan) and a lipid droplet (magenta) were manually segmented. Where the presence of a membrane was ambiguous a gap was left. Presented at 15 frames per second. Stills from this video are presented in figure 5.

Journal of Cell Science • Supplementary information

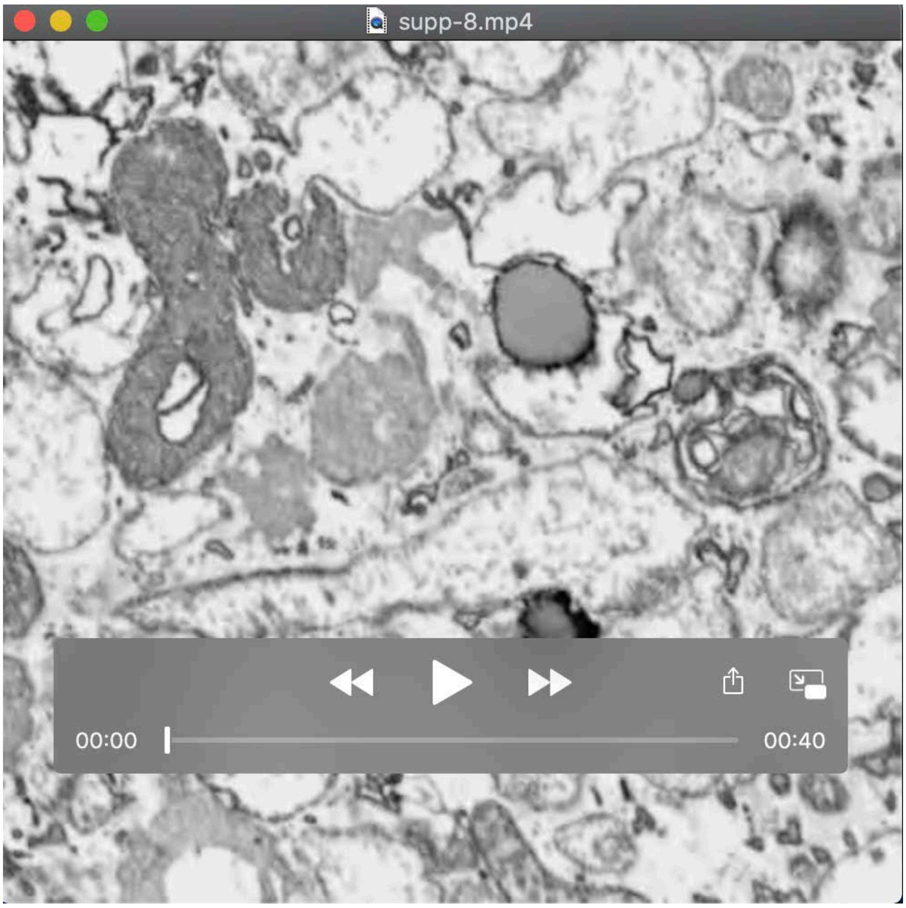

Movie 8. FIB SEM reconstruction of Mtb WT enwrapped in an LC3 positive membrane. 3D reconstruction of FIB SEM images obtained for ROI3 in figure 4. The limiting membrane of the bacteria (red), LC3 positive membranes (green), ER-like sheet of membrane (cyan and blue) and an intraluminal vesicle (magenta) were manually segmented. Where the presence of a membrane was ambiguous a gap was left. Presented at 15 frames per second. Stills from this video are presented in figure 5.
